# Systematic review and meta-analysis of deep learning for MSI-H in colorectal cancer whole slide images

**DOI:** 10.1038/s41746-025-01848-z

**Published:** 2025-07-18

**Authors:** Huo Li, Jing Qin, Zhongzhuan Li, Rong Ouyang, Zhixin Chen, Shijiang Huang, Shufen Qin, Qiliang Huang

**Affiliations:** 1 Department of Gastroenterology, The Fourth Affiliated Hospital of Guangxi Medical University, Liuzhou, China; 2 Department of General Medicine, Liuzhou People’s Hospital, Liuzhou, China

**Keywords:** Cancer, Gastrointestinal diseases, Gastrointestinal cancer, Colorectal cancer

## Abstract

This meta-analysis evaluated diagnostic performance of deep learning (DL) algorithms using whole slide images (WSIs) for detecting microsatellite instability-high (MSI-H) in colorectal cancer (CRC). PubMed, Embase, and Web of Science were searched until January 2025. Nineteen studies comprising 33,383 samples were included. Bivariate random-effects models calculated pooled sensitivity/specificity with 95% CIs. The revised QUADAS-2 tool was used for quality assessment. Pooled patient-based internal validation showed a sensitivity of 0.88 and specificity of 0.86, while external validation revealed higher sensitivity of 0.93 but lower specificity of 0.71. Image-based analysis showed similar accuracy. Meta-regression identified center, reference standard, and tile size as major sources of heterogeneity, with no significant differences observed between internal and external performance. Overall, DL algorithms demonstrate excellent sensitivity in detecting MSI-H; however, their lower specificity in external validation suggests overfitting and highlights the need for algorithm standardization to improve generalizability and clinical utility.

## Introduction

Colorectal cancer (CRC) is a major global malignancy, ranking third in incidence and second in cancer-related mortality, thus contributing significantly to the global disease burden^1^. An important genomic alteration associated with CRC is microsatellite instability (MSI), which arises from defects in the mismatch repair system and occurs in approximately 5–20% of CRC cases^2^. Notably, the prevalence of MSI is stage-specific; it exceeds 20% in stage II CRC but drops to less than 5% in more advanced stages^3^.

MSI tumors, characterized by a high tumor mutational burden driven by the MSI carcinogenic pathway, produce numerous immunogenic neoantigens and express immune checkpoints. Consequently, MSI has been identified as a favorable prognostic marker for stage II CRC, with failure to detect MSI potentially leading to unnecessary adjuvant chemotherapy^4,5^. Furthermore, MSI status predicts immunotherapy response, as studies show CRC patients with MSI respond more effectively to immune checkpoint inhibitors^6^. According to the National Comprehensive Cancer Network guidelines, MSI testing is recommended for all metastatic CRC patients^7^. Similarly, the European Society for Medical Oncology advocates MSI evaluation before immunotherapy, and the U.S. Food and Drug Administration has approved MSI as an indication for cancer immunotherapy^8,9^.

MSI detection methods include immunohistochemistry (IHC) targeting mismatch repair (MMR) proteins such as MLH1, PMS2, MSH2, and MSH6, and polymerase chain reaction (PCR) assays to identify microsatellite instability^9^. PCR commonly examines mononucleotide repeats like BAT-25 and BAT-26, along with dinucleotide markers. MSI-H is classified as MSI, while low microsatellite instability (MSI-L) is grouped with microsatellite stable (MSS). Although these methods are the standard for CRC classification, they are expensive, time-intensive, and show reduced sensitivity in samples with low tumor cell content. Both IHC and PCR rely on advanced equipment and skilled pathologists, presenting challenges in resource-limited settings. Furthermore, dMMR occurs in only 10–15% of CRC cases, reducing the cost-effectiveness of universal screening^10–12^. Thus, there is an urgent need for a more accessible, accurate, and cost-efficient detection method to improve dMMR and MSI testing strategies and support the advancement of precision medicine.

The introduction of whole slide images (WSIs) in digital pathology has advanced AI-assisted diagnostics by enabling high-resolution analysis and sharing of tissue samples. This innovation has improved cancer diagnosis, classification, and prognosis, enhancing clinical practice and personalized treatment^13–15^. AI advancements address key challenges in molecular pathology, including time-consuming and costly testing methods. Since 2019, growing evidence has demonstrated the ability of DL to accurately identify MSI and MSS status from hematoxylin and eosin (H&E)-stained whole slide images of CRC and other tumors^16–18^. The first automated, end-to-end DL-based MSI/dMMR detection model, developed by Kather et al. in 2019, achieved an area under the curve (AUC) of 0.84 in the TCGA cohort^18^. Subsequent studies using novel methodologies have reported improved AUC values ranging from 0.78 to 0.98^18^. Echle et al. developed a DL classifier with an AUC of 0.96 in external validation^16^. Mohsin Bilal et al. introduced a weakly supervised DL framework with three CNN, achieving an AUC of 0.98 in external cohorts^19^. Wagner SJ et al. implemented a transformer-based approach for effective mutation status prediction^20^. In 2022, these advancements led to the first commercial DL biomarker detection algorithm (MSIntuit, Owkin, Paris/New York) being approved for routine clinical use in Europe^21^.

In recent years, DL algorithm based on WSIs methods have been increasingly studied for predicting the MSI-H status of CRC. However, the predictive performance and reliability of these DL models vary widely, and their overall performance remains uncertain. Therefore, this systematic review aims to combined current findings and evaluate the predictive performance of histological models in diagnosing MSI-H in CRC.

## Results

### Study selection

**Fig. 1:**
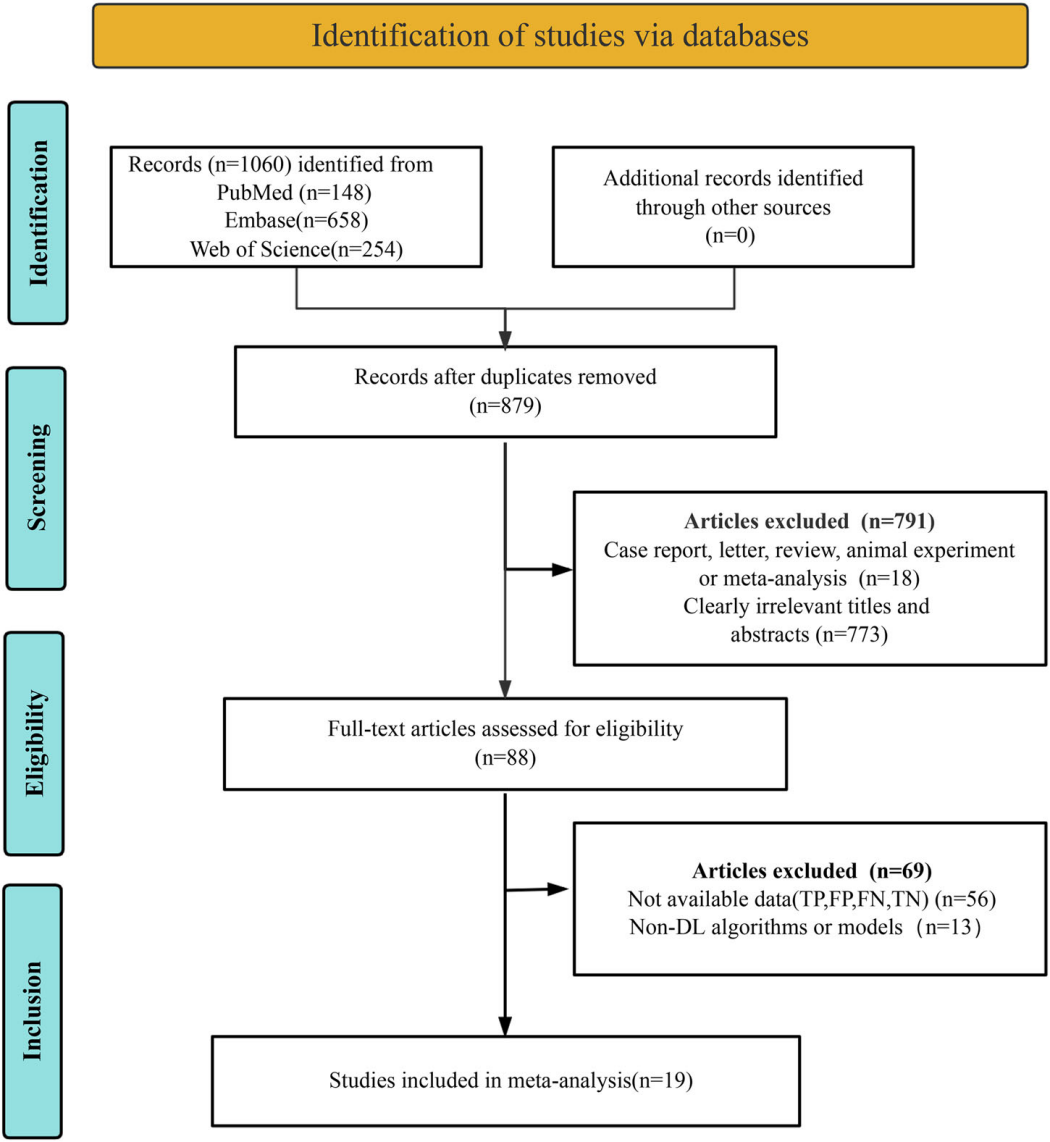
PRISMA flow diagram illustrating the study selection process. This figure presents a detailed overview of the systematic review process, including the number of studies identified, screened, and included at each stage.

The initial database search yielded 1060 potentially relevant articles. After removing 181 duplicates, 879 unique articles were subjected to preliminary screening. Strict application of inclusion criteria resulted in the exclusion of 791 articles. Following a detailed full-text review, 69 studies were further excluded due to insufficient or incomplete diagnostic data (TP, FP, FN, TN). Ultimately, 19 studies that met the criteria for evaluating the diagnostic performance of DL algorithms were included in the meta-analysis^16–18,21–36^. The literature screening process was systematically documented using a standardized Preferred Reporting Items for Systematic Reviews and Meta-Analyses (PRISMA) flow diagram, as shown in Fig. 1.

### Study description and quality assessment

A total of 19 eligible studies were included, with internal validation reported in 13 studies comprising 14 data sets and 14,324 patients or images (range: 100–4738). External validation was reported in 13 studies involving 19,059 patients or images (range: 35–2098), with 25 data sets. These studies were published between 2019 and 2024. All included studies were retrospective in design. Ten studies used PCR as the gold standard, while nine utilized a combination of PCR and IHC. The most commonly employed AI algorithms were CNN-based (10/19, 53%). A detailed summary of the study, patient, and technical characteristics is presented in Tables 1 and 2.

**Table 1:** Study and patient characteristics of the included studies

Author	Year	Center	Magnification	Tile Size	Analysis	Reference standard	Patients/images/per set			No. of MSI patients/images
							Training	Internal validation	External validation	
Hezi et al.	2024	Single	NR	NR	Patient-based	PCR	260	100	NR	Training:39 Internal validation:26
Gustav et al.	2024	Multiple	NR	224*224	Patient-based	PCR	2039	2039	429	Training:210 Internal validation:210 External validation:63
Tong et al.	2023	Single	NR	256*256	Patient-based	PCR/IHC	NR	646	111	Internal validation:323 External validation:54
Saillard et al.	2023	Multiple	40*	224*224	Image-based	PCR/IHC	NR	NR	1091	External validation:169
Niehues et al.	2023	Multiple	40*	256*256	Patient-based	PCR/IHC	NR	2190	2448	Internal alidation:245 External alidation:210
Guo et al.	2023	Multiple	40*	224*224	Patient-based	PCR	NR	NR	424	External alidation:61
Gerwert et al.	2023	Single	NR	256*256	Patient-based	PCR/IHC	331	147	NR	Training:142 Internal validation:26
Chang et al.	2023	Multiple	40*	512*512	Image-based	PCR	1579	1579	305	Training: Internal validation: External validation:
Qiu et al.	2022	Single	NR	224*224	Image-based	PCR	353	353	NR	Training:63 Internal validation:63
Wu et al.	2022	Multiple	20*	512*512	Patient-based	PCR/IHC	NR	441	696	Internal validation:77 External validation:127
Guo et al.	2022	Multiple	20*	512*512	Image-based	PCR/IHC	278	59	NR	Training:236 Internal validation:49
Fujii et al.	2022	Single	20*	256*256	Image-based	PCR	575	295	NR	Training:20 Internal validation:14
Echle et al.	2022	Multiple	20*	224*224	Patient-based	PCR/IHC	8343		9801	Training:1020 External validation:1231
Kather et al.	2019	Mutiple	NR	NR, NR	Patient-based	PCR	1053	NR	378	Training: NR External validation:28
Echle et al.	2020	Mutiple	20*	512*512	Patient-based	PCR/IHC	6046	6046	2302	Training:744 Internal validation:744 External validation:321
Cao et al.	2020	Mutiple	20*	512*512	Image-based	PCR	429	429	785	Training:71 Internal validation:71 External validation:164
Yamashita et al.	2021	Single	40*	256*256	Patient-based	PCR	100	NR	323	Training:50 Internal validation: NR External validation:52
Krause et al.	2021	Single	20*	512*512	Patient-based	PCR	NR	142	NR	Internal validation:21
Lee et al.	2021	Mutiple	20*	360*360	Patient-based	PCR/IHC	NR	NR	274	External validation:149

*Retro* retrospective, *Pro* prospective, *NR* not report, *WSIs* whole slide images, *PCR* polymerase chain reaction, *IHC* immunohistochemistry, *NGS* next-generation sequencing.

**Table 2:** Technical aspects of included studies

Author	Year	Optimal deep learning algorithm^a^	Interval validation sets				External validation sets			
			TP	FP	FN	TN	TP	FP	FN	TN
Hezi et al.	2024	Multiple Instance Learning (MIL)	22	10	4	64	NR	NR	NR	NR
Gustav et al.	2024	Transformer	83	219	27	1610	59	179	4	187
Tong et al.	2023	CNN	300	13	23	310	41	2	13	55
Saillard et al. (MAPTH-DP200)	2023	CNN	NR	NR	NR	NR	81	246	42	208
Saillard et al. (MAPTH-UFS)	2023	CNN	NR	NR	NR	NR	82	250	4	218
Niehues et al.	2023	Self-supervised, Attention-based Multiple-instance Learning	236	639	9	890	190	374	20	1455
Guo et al.	2023	Deep Learning (Swin Transformer using Shifted Windows)	NR	NR	NR	NR	52	95	9	329
Gerwert et al.	2023	CNN	22	19	4	102	NR	NR	NR	NR
Chang et al.	2023	Self-attention-enabled CNN	493	53	89	944	38	135	15	117
Qiu et al.	2022	CNN	47	75	16	215	NR	NR	NR	NR
Wu et al.(surgical)	2022	Multiple-instance Learning	70	18	7	346	80	13	8	254
Wu et al.(biopsy)	2022	Multiple-instance Learning	NR	NR	NR	NR	36	14	3	288
Guo et al.	2022	Deep Learning (Cascaded Network with Average Voting Ensemble)	35	1	14	9	NR	NR	NR	NR
Fujii et al. (second stage)	2022	CNN	6	19	0	80	NR	NR	NR	NR
Fujii et al. (2.5^th^ stage)	2022	CNN	7	49	1	148	NR	NR	NR	NR
Echle et al. (DACHS)	2022	CNN	NR	NR	NR	NR	205	1199	5	630
Echle et al. (DUESSEL)	2022	CNN	NR	NR	NR	NR	21	60	4	111
Echle et al. (MECC)	2022	CNN	NR	NR	NR	NR	94	403	12	174
Echle et al. (MUNICH)	2022	CNN	NR	NR	NR	NR	30	87	3	167
Echle et al. (NLCS)	2022	CNN	NR	NR	NR	NR	217	779	7	1095
Echle et al. (QUASAR)	2022	CNN	NR	NR	NR	NR	243	877	2	652
Echle et al. (TCGA)	2022	CNN	NR	NR	NR	NR	52	100	9	265
Echle et al. (UMM)	2022	CNN	NR	NR	NR	NR	3	6	1	25
Echle et al. (YORKSHIRE)	2022	CNN	NR	NR	NR	NR	111	519	1	102
Echle et al.(biopsy)	2022	CNN	NR	NR	NR	NR	211	1160	0	159
Kather et al.	2019	CNN	NR	NR	NR	NR	22	53	6	297
Echle et al.(surgical)	2020	CNN	638	924	105	4738	101	59	10	601
Echle et al.(biopsy)	2020	CNN	NR	NR	NR	NR	158	403	52	918
Cao et al.	2020	Ensemble (ResNet-18, GBDT, NB)	62	92	9	266	122	150	42	471
Yamashita et al.	2021	CNN	NR	NR	NR	NR	40	81	12	190
Krause et al.	2021	Generative Adversarial Network – GAN	16	30	5	91	NR	NR	NR	NR
Lee et al.	2021	CNN	NR	NR	NR	NR	139	8	10	117

*TP* true positive, *TN* true negative, *FP* false positive, *FN* false negative, *NR* not report.

*CNN* convolutional neural network, *RF* random forest.

^a^the algorithm with the highest area under the curve(AUC) value was extracted.

**Fig. 2:**
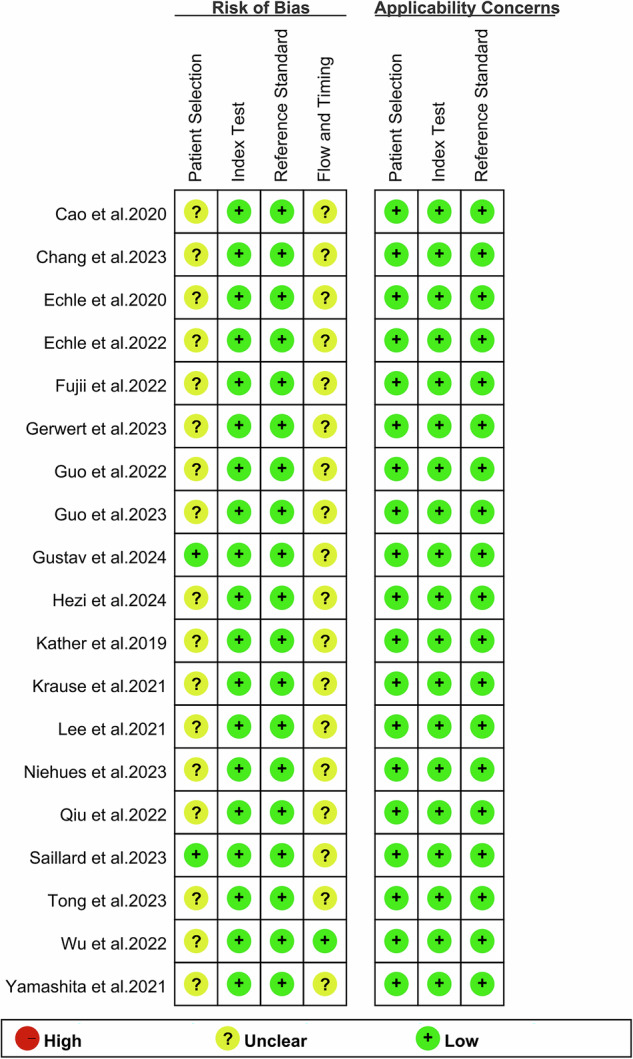
Risk of bias and applicability concerns of the included studies using the revised Quality Assessment of Diagnostic Performance Studies (QUADAS-2) tool. This figure was generated using Revman 5.4 software.

The risk of bias, assessed using the revised QUADAS-2 tool, is summarized in Fig. 2 and Supplementary Table 2. In the patient selection domain, 17 studies were rated as “unclear” due to insufficient information on whether patients were consecutively enrolled. Similarly, in the analysis domain, 17 studies were also rated as “unclear” because, although quality control and data filtering were mentioned, it was unclear whether all eligible patient samples were included in the analysis, raising concerns about potential analysis bias. Despite these limitations, the overall quality assessment indicates that the included studies are of acceptable quality, as most of the other items present low risks.

### Diagnostic performance of internal validation set for DL based on WSIs in predicting MSI-H in CRC patients in patient-based analysis

**Fig. 3:**
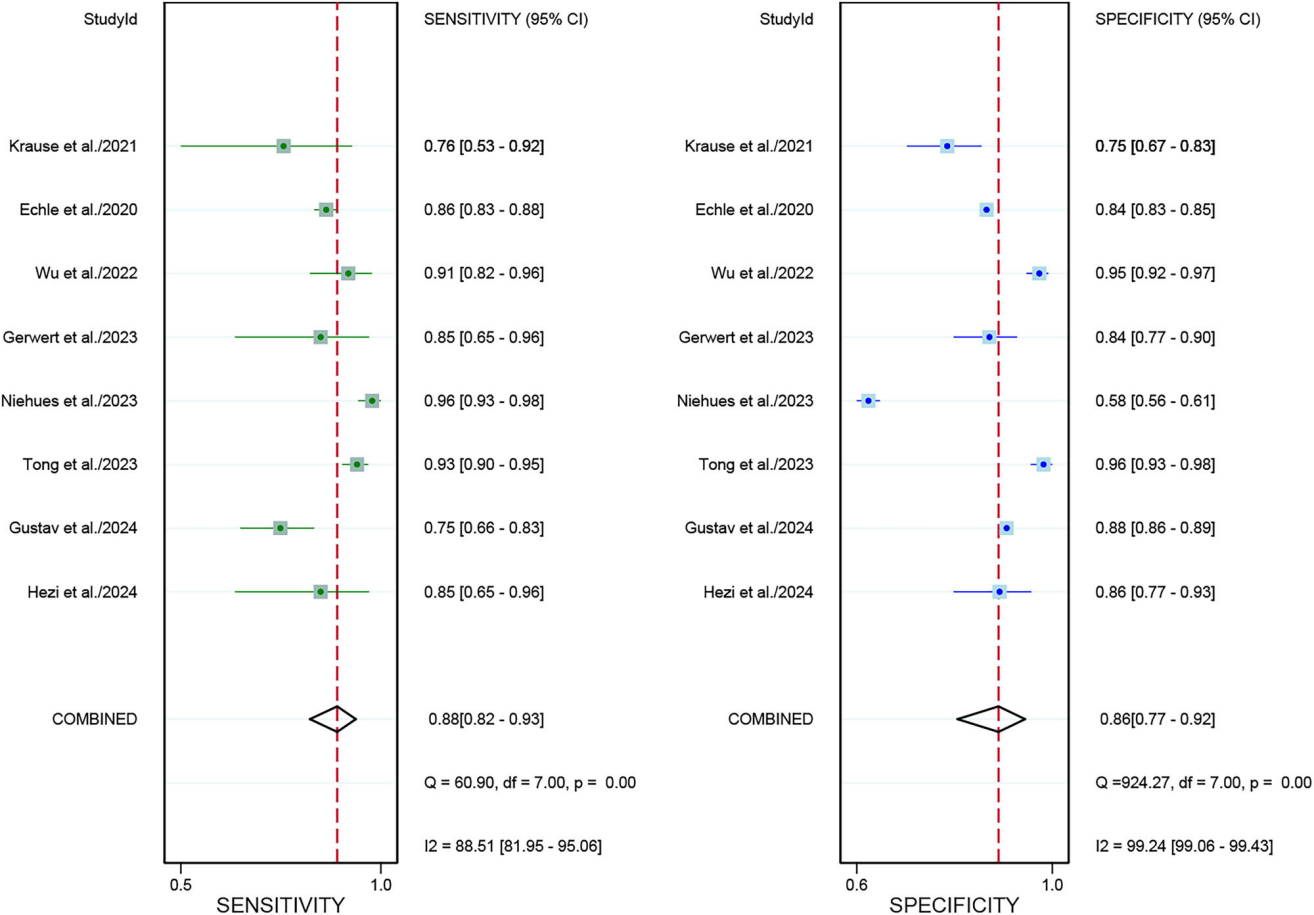
Forest plot of deep learning algorithms for identifying microsatellite instability-high in colorectal cancer using whole slide images in the internal validation set of patient-based analysis. Squares represent the sensitivity and specificity of each study, while horizontal bars indicate the 95% confidence intervals. This figure was generated using Stata 15.1 software.

**Fig. 4:**
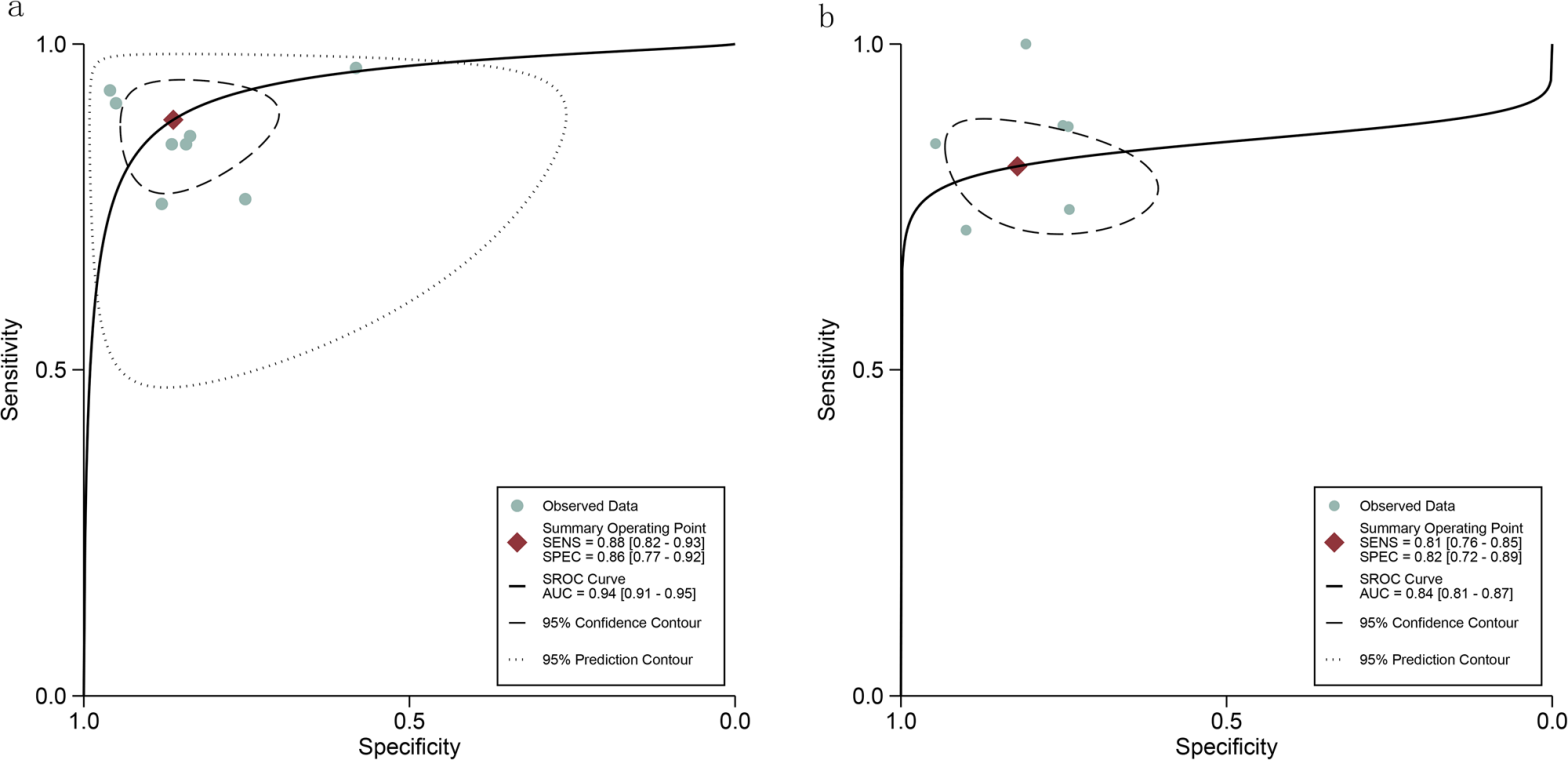
Summary receiver operating characteristic (SROC) curves of deep learning algorithms for identifying microsatellite instability-high (MSI-H) in colorectal cancer using whole slide images in the internal validation set. **a** Displays the patient-based SROC curve, indicating the diagnostic performance of the algorithms across different patients, while **b** provides the image-based SROC curve, reflecting the performance based on individual whole slide images.

**Fig. 5:**
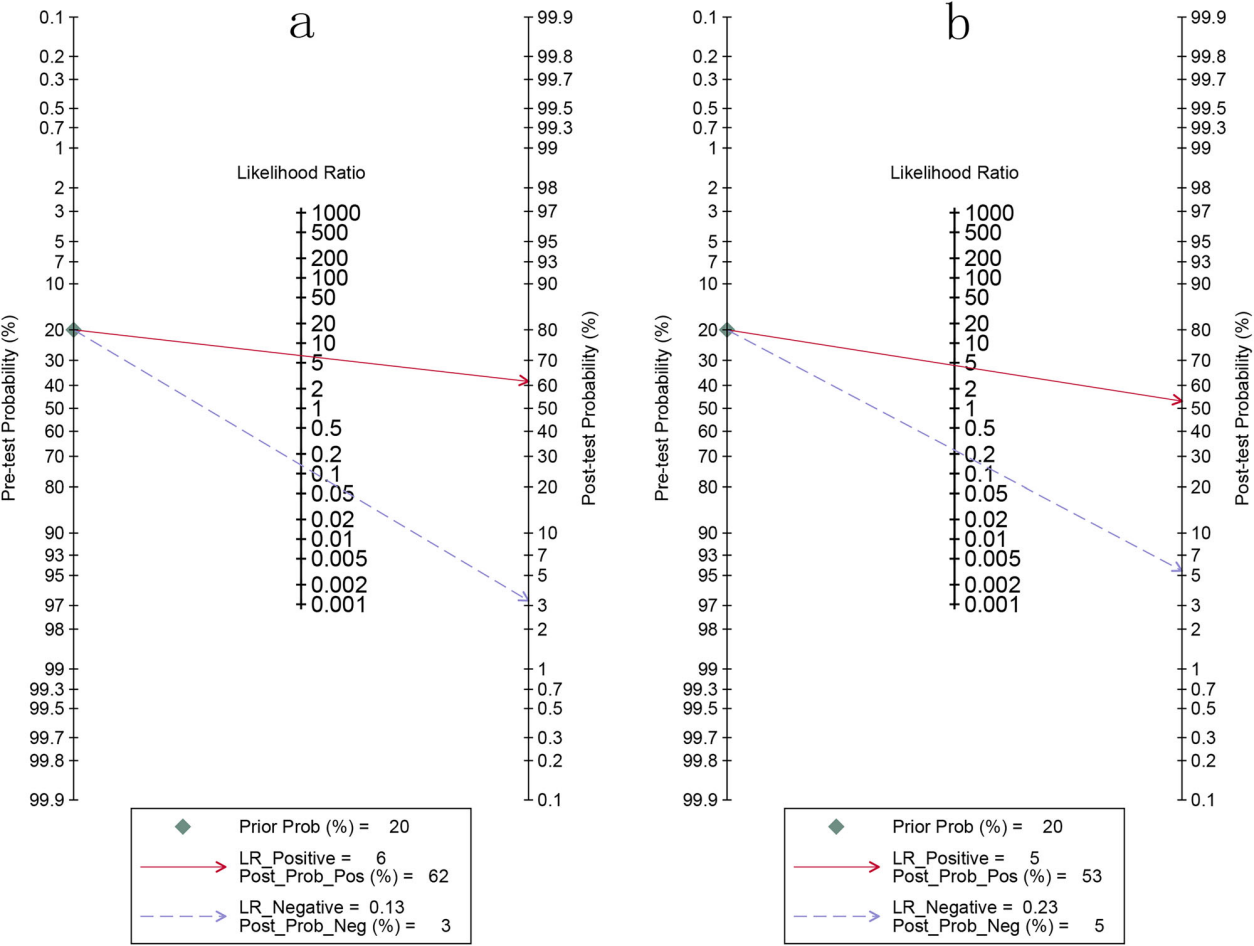
Fagan's nomogram for deep learning algorithms in identifying microsatellite instability-high (MSI-H) in colorectal cancer using whole slide images from the internal validation set. **a** Displays the patient-based nomogram, illustrating post-test probabilities of MSI-H classification based on pre-test probabilities and algorithm results. **b** Presents the image-based nomogram, assessing the likelihood of MSI-H based on individual whole slide imaging results.

For the internal validation dataset, DL algorithms based on WSIs achieved a sensitivity of 0.88 (95% CI: 0.82–0.93) and a specificity of 0.86 (95% CI: 0.77–0.92) in detecting MSI-H in CRC patients (Fig. 3). The AUC was 0.94 (95% CI: 0.91–0.95) (Fig. 4a). With a pre-test probability of 20%, the Fagan nomogram demonstrated a positive likelihood ratio of 62% and a negative likelihood ratio of 3% (Fig. 5a).

High heterogeneity was noted in sensitivity (I² = 88.51%) and specificity (I² = 99.24%) within the internal validation dataset. Meta-regression analysis identified that sensitivity heterogeneity was primarily driven by factors including center (single center vs. multicenter, *P* = 0.04), reference standard (Only PCR vs. non-only PCR, *P* < 0.001), and magnification (20× vs. 40×, *P* < 0.001). In the specificity heterogeneity analysis, no sources of heterogeneity related to the center, AI algorithm, reference standard, magnification, and tile size were found (all *P* > 0.05) (Table 3). The sensitivity analysis revealed no potential source of heterogeneity (Supplementary Table 3).

**Table 3:** Meta-regression analysis of deep learning algorithm performance based on patient-based analysis in internal validation cohorts for diagnosing microsatellite instability-high (MSI-H) in colorectal cancer using whole slide images

Subgroup	Studies, *n*	Sensitivity(95%CI)	Meta-regression *P*-value	Specificity(95%CI)	Meta-regression *P*-value
**Center**			0.04		0.40
Single center	4	0.87 (0.79–0.96)		0.88 (0.79–0.97)	
Multi-center	4	0.89 (0.83–0.95)		0.85 (0.74–0.95)	
**Optimal AI algorithm**			0.08		0.82
CNN	3	0.89 (0.81–0.97)		0.90 (0.81–0.98)	
Non-CNN	5	0.86 (0.81–0.95)		0.84 (0.74–0.94)	
**Reference standard**			<0.001		0.16
Only PCR	3	0.79 (0.68–0.89)		0.84 (0.71–0.97)	
non-only PCR	5	0.91 (0.88–0.95)		0.87 (0.79–0.96)	
**Tile size**			0.25		0.35
224*224 or 256*256	4	0.90 (0.84–0.96)		0.86 (0.74–0.97)	
512*512	3	0.86 (0.77–0.96)		0.87 (0.75–0.99)	
**Magnification**			<0.001		0.15
20*	3	0.87 (0.82–0.92)		0.87 (0.78–0.95)	
40*	1	0.96 (0.93–0.99)		0.58 (0.28–0.88)	

*CNN* Convolutional Neural Network, *PCR* polymerase chain reaction.

### Diagnostic performance of internal validation set for DL based on WSIs in predicting MSI-H in CRC patients in image-based analysis

**Fig. 6:**
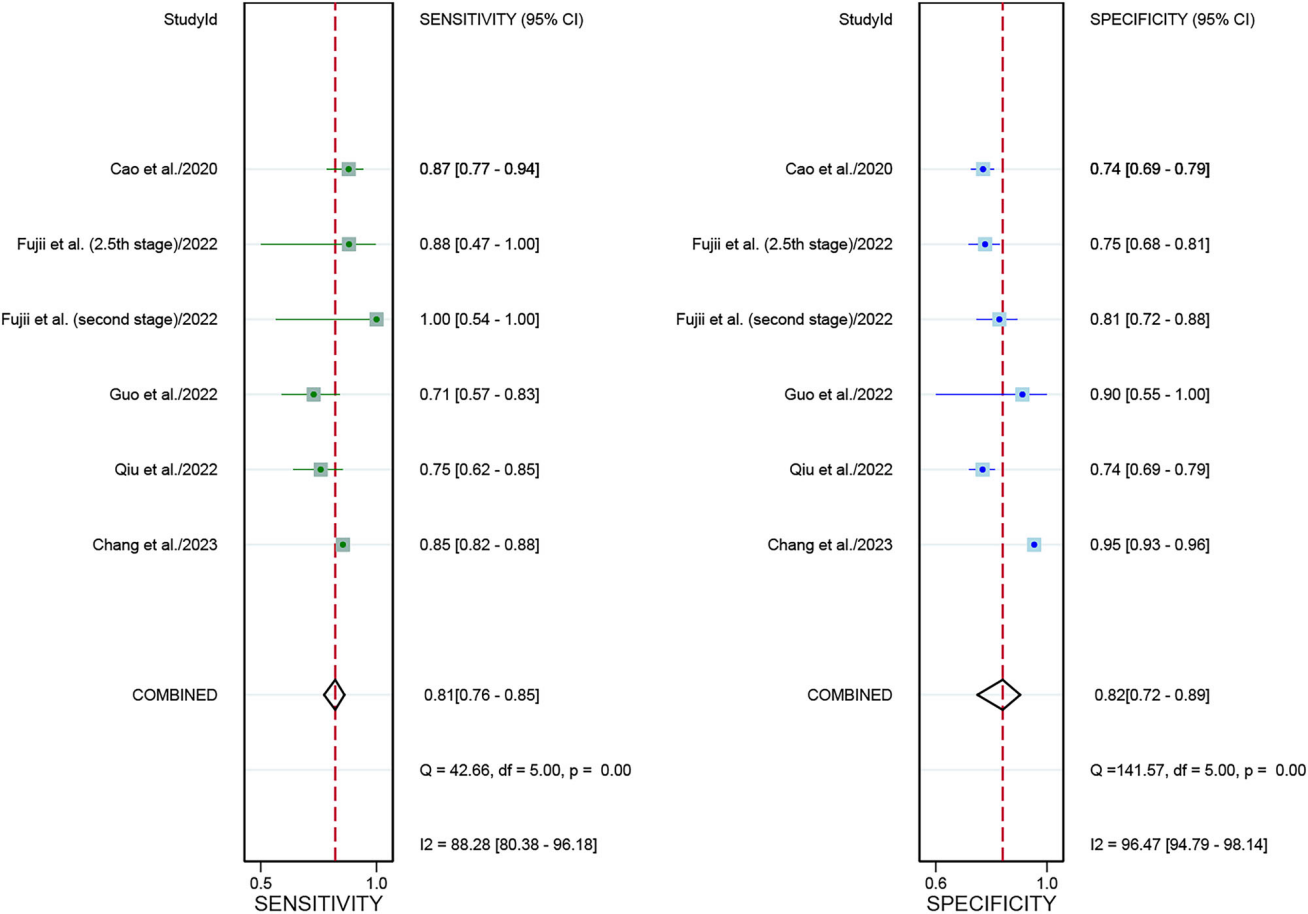
Forest plot of deep learning algorithms for identifying microsatellite instability-high in colorectal cancer using whole slide images in the internal validation set of image-based analysis. Squares represent the sensitivity and specificity of each study, while horizontal bars indicate the 95% confidence intervals. This figure was generated using Stata 15.1 software.

For the internal validation dataset, DL algorithms based on WSIs achieved a sensitivity of 0.81 (95% CI: 0.76–0.85) and a specificity of 0.82 (95% CI: 0.72–0.89) in detecting MSI-H in CRC patients (Fig. 6). The AUC was 0.84 (95% CI: 0.81–0.87) (Fig. 4b). With a pre-test probability of 20%, the Fagan nomogram demonstrated a positive likelihood ratio of 53% and a negative likelihood ratio of 5% (Fig. 5b).

High heterogeneity was noted in sensitivity (I² = 88.28%) and specificity (I² = 96.47%) within the internal validation dataset. The sensitivity analysis revealed that after Omitting Chang et al. the I^2^ for sensitivity was 17.16%, for specificity was 0%, suggesting it was the potential source of heterogeneity (Supplementary Table 3).

### Diagnostic performance of external validation sets for DL based on WSIs in predicting MSI-H in CRC patients in patient-based analysis

For the external validation dataset, the sensitivity of detecting MSI-H in CRC was 0.93 (95% CI: 0.88–0.95), while the specificity was 0.71 (95% CI: 0.57–0.82) (Supplementary Fig. 1). The AUC was 0.92 (95% CI: 0.90–0.94) (Supplementary Fig. 2a). At a pre-test probability of 20%, the Fagan nomogram indicated a positive likelihood ratio of 44% and a negative likelihood ratio of 3% (Supplementary Fig. 3a).

High heterogeneity was identified for sensitivity (I² = 95.30%) and specificity (I² = 99.59%) within the external validation dataset. Meta-regression analysis revealed that the heterogeneity in sensitivity was primarily influenced by the center (single center vs. multicenter), reference standard (Only PCR vs. non-only PCR), (*P* = 0.03, *P* < 0.001) while that in specificity was mainly driven by tile size (256*256 or 224*224 vs. 512*512) (*P* < 0.001) (Table 4). The sensitivity analysis revealed no potential source of heterogeneity (Supplementary Table 4).

**Table 4:** Meta-regression analysis of deep learning algorithm performance based on patient-based analysis in external validation cohorts for diagnosing microsatellite instability-high (MSI-H) in colorectal cancer using whole slide images

Subgroup	Studies, *n*	Sensitivity(95%CI)	Meta-regression *P*-value	Specificity(95%CI)	Meta-regression *P*-value
**Center**			0.03		0.21
Single center	2	0.77 (0.51–0.1.00)		0.88 (0.66–1.00)	
Multi-center	19	0.93 (0.90–0.97)		0.68 (0.55–0.82)	
**Optimal AI algorithm**			0.23		0.06
CNN	16	0.93 (0.89–0.97)		0.65 (0.50–0.80)	
Non-CNN	5	0.91 (0.83–1.00)		0.80 (0.69–1.00)	
**Reference standard**			<0.001		0.23
Only PCR	7	0.89 (0.80–0.98)		0.62 (0.37–0.86)	
non-only PCR	14	0.94 (0.91–0.98)		0.75 (0.61–0.89)	
**Tile size**			0.87		<0.001
224*224 or 256*256	15	0.94 (0.90–0.98)		0.58 (0.44–0.72)	
512*512	4	0.88 (0.75–1.00)		0.91 (0.81–1.00)	
**Magnification**			0.71		0.53
20*	15	0.95 (0.91–0.98)		0.66 (0.50–0.82)	
40*	3	0.86 (0.70–1.00)		0.76 (0.47–1.00)	

*CNN* Convolutional Neural Network, *PCR* polymerase chain reaction.

There was no statistically significant difference in sensitivity, specificity and AUC values between the internal and external validation datasets in patient-based analysis (Z = –1.50, 0.67,1.39; *P* = 0.13, 0.50,0.17).

### Diagnostic performance of external validation sets for DL based on WSIs in predicting MSI-H in CRC patients in image-based analysis

For the external validation dataset, the sensitivity of detecting MSI-H in CRC was 0.80 (95% CI: 0.63–0.90), while the specificity was 0.54 (95% CI: 0.41–0.67) (Supplementary Fig. 4). The AUC was 0.71 (95% CI: 0.66–0.74) (Supplementary Fig. 2b). At a pre-test probability of 20%, the Fagan nomogram indicated a positive likelihood ratio of 30% and a negative likelihood ratio of 9% (Supplementary Fig. 3b). The sensitivity analysis revealed that after Omitting Saillard et al. (MAPTH-UFS), the I^2^ for sensitivity was 31.30%, suggesting it was the potential source of heterogeneity (Supplementary Table 4).

There was no statistically significant difference in sensitivity values between the internal and external validation datasets in image-based analysis (Z = 0.14; *P* = 0.89). However, the specificity and AUC of the internal validation dataset was significantly higher than that of the external validation dataset (Z = 3.53,5.10; both *P* < 0.001).

### Publication bias

**Fig. 7:**
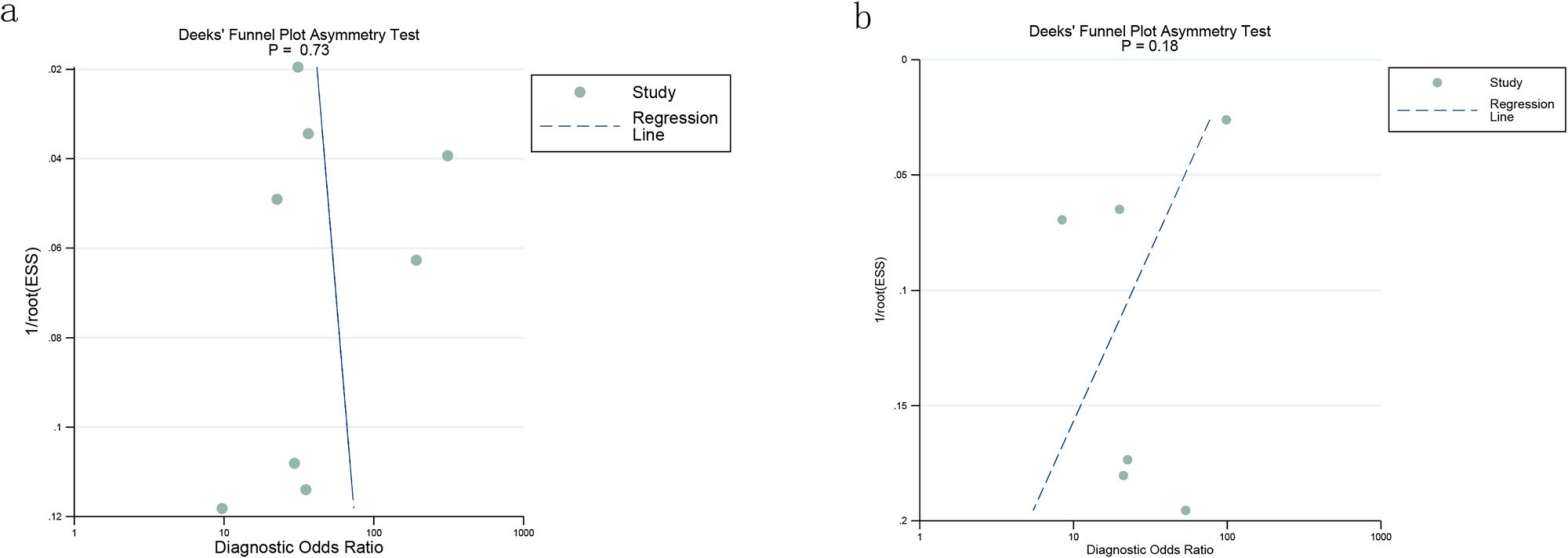
Deek's funnel plot of internal validation set. **a** Presents the patient-based analysis, while **b** illustrates the image-based analysis. The funnel plot visually represents the relationship between study size and treatment effect, helping to assess publication bias. Asymmetry in the plots may indicate potential biases in the included studies. A *P* value of 0.05 was considered significant in evaluating this bias. This figure was generated using Stata 15.1 software.

The Deeks’ funnel plot asymmetry test showed no significant publication bias in the internal validation dataset based on patient-based and image-based analyses for DL (*P* = 0.73, *P* = 0.18) (Fig. 7). Likewise, no significant publication bias was detected in the external validation dataset (*P* = 0.80, *P* = 0.77) (Supplementary Fig. 5).

## Discussion

To the best of our knowledge, this is the first meta-analysis to evaluate the diagnostic performance of DL algorithms in detecting MSI-H in CRC using WSIs. For the internal validation dataset, the patient-based analysis yielded a sensitivity of 0.88 and a specificity of 0.86, while the image-based analysis showed a sensitivity of 0.81 and a specificity of 0.82. The AUC for sensitivity was 0.94 and for specificity was 0.84. In contrast, the external validation dataset demonstrated a higher sensitivity of 0.93 and a specificity of 0.71 in the patient-based analysis. The image-based analysis for the external dataset revealed a sensitivity of 0.80 and a specificity of 0.54. The AUC was 0.92 for the patient-based analysis and 0.71 for the image-based analysis. These results suggest that while DL algorithms effectively identify MSI-H in CRC, their performance varies between internal validation and external validation datasets. The outstanding diagnostic performance of deep learning algorithms can be attributed to their ability to automatically learn complex morphological features associated with MSI-H directly from digital pathology slides, features that conventional pathologists may overlook with the naked eye^37^. The higher specificity in internal validation datasets likely results from consistent data preprocessing, uniform staining, and standardized image acquisition, which help the model accurately distinguish MSI-H from non-MSI-H cases. In contrast, external validation datasets often introduce greater variability due to differences in staining protocols, slide preparation, and image quality, leading to domain shifts and reduced specificity^38^. These findings highlight the need for standardized data pipelines and the inclusion of multi-center datasets to enhance generalizability. Although DL demonstrates significant potential for MSI-H detection, caution is warranted due to dataset-specific factors and the absence of standardized external validation protocols, which may introduce bias. Future studies should focus on collaborative frameworks to develop robust and diverse training datasets while adopting cross-validation strategies to mitigate overfitting and improve clinical applicability^39^.

In terms of internal and external validation datasets revealed that patient-based approaches demonstrated higher sensitivity compared to image-based analysis (0.88 vs. 0.83, 0.92 vs. 0.80). In patient-based methods, each patient is represented by one WSI image as an independent sample, whereas image-based methods may include multiple slices from the same patient. Independent sampling ensures the model captures a broader range of variability, enhancing its predictive performance across diverse patient populations^40^. Patient-based approaches reflect greater diversity, encompassing variations in tumor types, stages, and therapeutic responses. This diversity improves the model’s generalizability by enabling it to learn a wider range of features, including tumor staging and demographic characteristics^41^. In contrast, image-based training risks overfitting to specific features within individual patients, which may limit the model’s applicability to external datasets^42^.

In the internal and external validation of AI algorithms, meta-regression analysis revealed no significant statistical differences in sensitivity or specificity between the patient-based CNN and non-CNN groups. For non-CNN models, for instance, Niehues’ study demonstrated that a self-supervised, attention-based multiple-instance learning model effectively focused on relevant tissue regions^24^. Visualization of the attention mechanism revealed that, for MSI prediction, the model concentrated primarily on tumor tissues while minimally focusing on fibromuscular and non-tumor epithelium. However, some attention dispersion was observed, potentially contributing to the finding that attention-augmented models did not outperform standalone CNN algorithms in sensitivity or specificity. Future comparisons of the diagnostic performance among different deep learning algorithms is a promising area for exploration.

It should be noted that in our patient-based external validation dataset, larger tiles (512*512) demonstrated higher specificity compared to smaller tiles (224*224 or 256*256) (0.91 vs. 0.58, *P* < 0.001). Larger tiles enhance the model’s ability to capture localized features, which is critical for identifying subtle pathological changes. Conversely, while smaller tiles can provide broader contextual information, they may overlook key details^43,44^. Although DL algorithms offer promise for improving pathological diagnosis, further research is needed to explore the impact of tile size on model performance and to ensure the reliability of clinical applications.

Furthermore, we found that in the meta-regression analysis using the reference standard, patient-based internal and external validation showed that the sensitivity of the non-only PCR group was significantly higher than that of the only PCR group. But current evidence indicates that PCR demonstrates greater diagnostic performance than IHC as a reference standard for identifying MSI in CRC, especially regarding sensitivity and specificity^45,46^. Since PCR has higher specificity than IHC for detecting MSI-H, using IHC as the gold standard results in a higher false positive rate (i.e., cases deemed positive by IHC that are not truly positive). In this situation, as long as the deep learning model detects any morphological features associated with IHC positivity in the images, these cases will be counted as “true positives,” thus overestimating the model’s sensitivity. In contrast, when PCR is used as the reference standard, the model is required to accurately identify PCR-positive cases. Although this may decrease sensitivity, it offers a more precise reflection of the actual biological state. Nonetheless, Heterogeneity among studies and the relatively small number of articles in the only-PCR group may contribute to potential instability in the results. Therefore, future research involving larger sample sizes is essential to evaluate the diagnostic performance of different reference standards and achieve more robust findings.

While previous systematic reviews, such as those by Davri et al.^47^ and Guitton et al.^48^, have offered valuable insights into the use of DL for CRC diagnosis and the prediction of MSI from WSIs, our study enhances this foundation by incorporating a broader range of internal and external datasets for systematic statistical analysis. This approach improves the assessment of the model’s adaptability across varied populations. Additionally, we emphasize the necessity of standardizing algorithms to mitigate potential overfitting issues during external validation, a concern that has not been thoroughly addressed in existing literature.

Compared to the previous meta-analysis by Ying et al. and Alam et al., our meta-analysis is the first to predict MSI-H in CRC using WSIs. Our study also includes a larger sample size and incorporates more studies. Ying et al.’s meta-analysis used complex confounding models, combining traditional machine learning, clinical, and genomic features, leading to limited scalability^49^. Alam et al.’s study evaluated MSI prediction across multiple cancer types, including colorectal, gastric, ovarian, and endometrial cancers, but did not perform a pooled analysis of DL’s diagnostic performance specifically for MSI-H in CRC^50^. In another meta-analysis, Wang et al. assessed AI-based radiomics for MSI prediction in CRC but included fewer studies(14 studies) and limited external validation datasets (four datasets)^51^.Their reported AUC was 0.83 and sensitivity was 0.76, both lower compared to our AUC of 0.90 and sensitivity of 0.91. Moreover, nine out of 12 studies in Wang et al.’s analysis relied on PET/CT, which is expensive and diverges from AI’s goal of cost-effective diagnostics. In contrast, our study demonstrates that AI models based on WSIs can efficiently identify MSI-H in CRC, providing new evidence for their clinical applicability and advantages in CRC diagnosis.

The high heterogeneity among the included studies may have influenced the pooled sensitivity and specificity of DL in both internal and external validation datasets. Multiple meta-regression identified center, AI algorithm, analysis method, magnification, tile size, and reference standard as sources of heterogeneity in internal validation sensitivity. For external validation sensitivity, analysis method, magnification, and tile size were key contributors. In specificity, center, AI algorithm, tile size, and reference standard influenced internal validation, while magnification was the sole factor in external validation. However, this heterogeneity may stem from other potential factors such as clinical staging of colorectal cancer, dataset size, regional populations, WSI image quality, and specimen origin (e.g., surgical resection or endoscopic biopsy).

Our results demonstrate that DL-based methods achieve high diagnostic performance for MSI-H detection in colorectal cancer across both internal and external datasets. AI has the potential to reduce clinicians’ workloads, minimize diagnostic errors, and prevent adverse outcomes associated with misdiagnoses. However, only one study in our analysis directly compared AI to human performance. Kather et al. reported a sensitivity and specificity of 0.5 for pathologists^18^. Future studies should focus on comparative evaluations between AI and human performance, particularly that of pathologists. Beyond diagnostic performance, cost-effectiveness is crucial for integrating AI models into routine practice. In hypothetical metastatic CRC populations, combining high-sensitivity AI with confirmatory MSI testing could save approximately $400 million^52^. AI models also expedite treatment initiation, reducing average time to less than a day and improving patient outcomes. Once trained, AI systems require minimal maintenance costs, while offering valuable insights that may reduce unnecessary treatments or accelerate diagnoses^52^. Despite these promising potentials, several challenges remain. AI models require large, diverse datasets for robust validation and effective integration into routine clinical workflows. Training these models is time-consuming, often needing hundreds or thousands of annotated images, which may involve extensive manual labeling. Moreover, concerns regarding data privacy, model interpretability, and regulatory approval further complicate implementation. Addressing these challenges is essential to ensure the successful and safe adoption of AI in clinical practice^50^.

Several limitations of this meta-analysis warrant careful consideration when interpreting the results. First, the training and validation cohorts for all included models were retrospective, which may introduce potential bias. Prospective studies are needed to validate these findings and ensure their applicability in clinical practice^23^. Second, some studies used a combination of PCR and IHC as the reference standard. Weak staining in IHC could result in missed cases, potentially biasing the diagnostic performance for identifying MSI-H in CRC^53^. Third, model training heavily relied on specific open datasets (e.g., TCGA, QUASAR, DACHS), with limited use of local clinical WSIs images for training and validation. This reliance may lead to bias and hinder the assessment of the model’s generalizability. Fourth, we recognize that selecting only the highest-performing algorithm from multi-model studies may introduce positive performance bias, as it does not represent the full range of tested algorithms. To minimize patient overlap among the included studies, we chose to extract only the best-performing algorithm from each study, which may lead to an overestimation of performance. Furthermore, due to limited data availability, we used estimated maximum Youden indices, which could also contribute to bias in performance estimates. It is also important to highlight that the QUADAS-2 assessment revealed “unclear” risk of bias in patient selection for 17 out of 19 studies and analysis domains for 18 out of 19 studies, indicating potential spectrum bias and selective reporting.

In conclusion, this meta-analysis confirms that DL algorithms perform excellently in detecting microsatellite MSI-H in CRC using WSIs. However, their lower specificity in external validation suggests overfitting and highlights the need for algorithm standardization to improve generalizability and clinical utility.

## Methods

This meta-analysis was conducted in full compliance with the Preferred Reporting Items for Systematic Reviews and Meta-Analyses of Diagnostic Test Accuracy (PRISMA-DTA) guidelines^54^. Additionally, the study protocol has been registered in the PROSPERO database (CRD42025632819).

### Search strategy

We conducted a systematic literature search using the PubMed, Embase, and Web of Science databases, with the initial search completed on December 15, 2024. A second search was conducted in January 2025 to include newly published studies. The search strategy involved three groups of keywords: artificial intelligence-related terms (e.g., artificial intelligence, machine learning, deep learning), target-related terms (e.g., microsatellite instability, dMMR), and disease-specific terms (e.g., colon cancer, rectal cancer, colorectal cancer). Both free-text keywords and Medical Subject Headings (MeSH) terms were used to ensure precision. Detailed search strategies are available in Supplementary Table 1. Additionally, the references of included studies were reviewed to identify additional relevant literature.

### Inclusion and exclusion criteria

These studies were carefully selected following the PITROS framework. Participants (P): The participants in this study are patients diagnosed with CRC. Index test (I): This study employs DL techniques to analyze WSIs for predicting MSI-H. Target condition (T): The positive group is defined as patients with high MSI-H, while the negative group is defined as patients with MSS or MSI-L. Reference standard (R): The reference standard is PCR or IHC to validate the accuracy of the MSI status. Outcomes (O): The primary outcomes include sensitivity, specificity, and the AUC. Setting (S): The study setting includes retrospective or prospective data sources, covering public databases or local hospitals.

Exclusion criteria included studies on animals, non-original articles (e.g., reviews, case reports, conference abstracts, meta-analyses, and letters to editors), and non-English publications due to accessibility issues. Furthermore, studies using general artificial intelligence approaches that are unrelated to deep learning algorithms, such as classic machine learning techniques (e.g., support vector machines (SVM), logistic regression (LR), and random forests (RF)), were excluded. Additionally, studies that relied solely on non-AI methods, such as those using WSIs for diagnosis without employing any AI algorithms, were also excluded.

### Quality assessment

To ensure a rigorous assessment of the quality of the included studies, we revised the Quality Assessment of Diagnostic Accuracy Studies-2 (QUADAS-2) tool. Irrelevant criteria were replaced with standards better suited to evaluating the risk of bias in predictive models. This section outlines modifications made to the tool, informed by experience with the original framework and potential sources of bias arising from variations in study design and implementation.

The revised QUADAS-2 tool includes four domains: patient selection, index test (AI algorithms), reference standard, and analysis. Bias was evaluated across all four domains, while applicability concerns were assessed for the first three. Two reviewers (HL and ZZ) independently applied the modified tool to assess the risk of bias in the included studies, resolving any disagreements through discussion to reach consensus.

### Data extraction

Two independent reviewers (HL and JQ) screened the titles and abstracts of the remaining articles to identify potentially eligible studies, with a third reviewer (OY) serving as an arbitrator to resolve any disagreements. Extracted data included the first author’s name, study type, publication year, country of data origin, number of study centers, and patients and images data for the training, internal validation, and external validation sets (e.g., number of enrolled patients, number of images reference standard, diagnostic model algorithm, statistical analysis method, tile size, and magnification). For studies lacking data required for meta-analysis, we contacted corresponding authors via email to request the missing information.

In cases where diagnostic contingency 2×2 tables were not provided, we employed two strategies to construct them: (1) calculating the number of true positives (TP) and total cases based on sensitivity, specificity, and the reference standard; and (2) extracting optimal sensitivity and specificity from ROC curve analyses using the Youden index.

### Outcome measures

The primary outcome measures were sensitivity, specificity, and the AUC for internal and external validation sets. Sensitivity, also known as recall or the true positive rate, measures the probability of correctly identifying true MSI-H cases and is calculated as TP/ (TP+ false negative (FN)). Specificity, or the true negative rate, reflects the probability of correctly identifying MSS or MSI-L cases and is calculated as true negative (TN)/(TN+ false positive (FP)). AUC, representing the area under the ROC curve, provides a comprehensive metric of the model’s ability to distinguish between positive and negative cases. For studies presenting multiple contingency tables based on different datasets or types of colorectal cancer, we assumed independence and extracted all contingency tables. Additionally, for studies evaluating multiple deep learning models, only the model with the highest AUC from the internal or external validation sets was extracted.

### Statistical analysis

This study utilized a bivariate random-effects model for the meta-analysis to assess the diagnostic performance of deep learning in predicting MSI-H in CRC using WSIs. Sensitivity and specificity were pooled separately for internal and external validation sets. Forest plots visually presented the pooled sensitivity and specificity, while a summary receiver operating characteristic (SROC) curve provided pooled estimates with 95% CIs and prediction intervals. Heterogeneity across studies was evaluated using Higgins’ I² statistic, with I² values of 25%, 50%, and 75% indicating low, moderate, and high heterogeneity, respectively^55^. Meta-regression analyses were conducted to identify sources of significant heterogeneity (I² > 50%)^56^.Meta-regression variables included AI algorithm type (CNN, non-CNN), analysis type (patient-based, image-based), reference standard (only PCR, not only PCR), tile size (256 × 256 or 224 × 224, 512 × 512), magnification (20×, 40×), and study center type (single, multiple). Univariate subgroup analyses were performed for these variables, with statistical differences between subgroups evaluated using the likelihood ratio test.

Potential publication bias was assessed using Deeks’ funnel plot asymmetry test^57^. Statistical analyses were conducted with the Midas and Metadat modules in Stata version 15.1, while RevMan 5.4 from the Cochrane Collaboration was used for risk of bias assessment. All statistical tests were two-sided, with *P* < 0.05 considered statistically significant, and results were reported with 95% confidence intervals.

## Supplementary information

Supplementary materials

## Data Availability

All data generated or analyzed during this study are included in this published article. Further inquiries can be directed to the corresponding author.

## Code availability

Not applicable. No custom code was generated or used in this study.
