# Supplementary material for: Systematic review and meta-analysis of deep learning for MSI-H in colorectal cancer whole slide images
Source: NPJ Digit Med. 2025 Jul 18;8:456. doi: 10.1038/s41746-025-01848-z (PMC12274608; doi:10.1038/s41746-025-01848-z)

Preferred Reporting Items for Systematic Reviews and Meta-Analyses of Diagnostic Test Accuracy (PRISMA-DTA) Checklist.

**TITLE/ABSTRACT**			Reported on page #
Title	1	Identify the report as a systematic review (+/−meta-analysis) of diagnostic test accuracy (DTA) studies	1
Abstract	2	See the PRISMA for Abstracts checklist.	1-2
**INTRODUCTION**			
Rationale	3	Describe the rationale for the review in the context of what is already known	2
Clinical role of index test	D1	State the scientific and clinical background, including the intended use and clinical role of the index test, and if applicable, the rationale for minimally acceptable test accuracy (or minimum difference in accuracy for comparative design)	2
Objectives	4	Provide an explicit statement of question(s) being addressed in terms of participants, index test(s), and target condition(s)	3
**METHODS**			
Protocol and registration	5	Indicate if a review protocol exists, if and where it can be accessed (e.g., Web address), and, if available, provide registration information including registration number.	11
Eligibility criteria	6	Specify study characteristics (participants, setting, index test(s), reference standard(s), target condition(s), and study design) and report characteristics (e.g., years considered, language, publication status) used as criteria for eligibility, giving rationale.	11-12
Information sources	7	Describe all information sources (e.g., databases with dates of coverage, contact with study authors to identify additional studies) in the search and date last searched.	11
Search	8	Present full search strategies for all electronic databases and other sources searched, including any limits used, such that they could be repeated.	11

Selection process	9	State the process for selecting studies (i.e., screening, eligibility, included in systematic review, and, if applicable, included in the meta-analysis).	11
Data collection process	10	Describe method of data extraction from reports (e.g., piloted forms, independently, in duplicate) and any processes for obtaining and confirming data from investigators.	12-13
Definitions for data extraction	11	Provide definitions used in data extraction and classifications of target condition(s), index test(s), reference standard(s) and other characteristics (e.g., study design, clinical setting).	11-12
Risk of bias and applicability	12	Describe methods used for assessing risk of bias in individual studies and concerns regarding the applicability to the review question.	12
Diagnostic accuracy measures	13	State the principal diagnostic accuracy measure(s) reported (e.g., sensitivity, specificity) and state the unit of assessment (e.g., per-patient, per-lesion).	13
Synthesis of results	12	Describe methods of handling data, combining results of studies and describing variability between studies. This could include, but is not limited to: a) handling of multiple definitions of target condition. b) handling of multiple thresholds of test positivity, c) handling multiple index test readers, d) handling of indeterminate test results, e) grouping and comparing tests, f) handling of different reference standards	13-14
Meta-analysis	D2	Report the statistical methods used for meta-analyses, if performed.	13-14
Additional analyses	16	Describe methods of additional analyses (e.g., sensitivity or subgroup analyses, meta-regression), if done, indicating which were pre-specified.	14
**RESULTS**			
Study selection	17	Provide numbers of studies screened, assessed for eligibility, included in the review (and included in meta-analysis, if applicable) with reasons for exclusions at each stage, ideally with a flow diagram.	3-4
Study	18	For each included study provide citations and present key characteristics including: a) participant characteristics	4

characteristics		(presentation, prior testing), b) clinical setting, c) study design, d) target condition definition, e) index test, f) reference standard, g) sample size, h) funding sources	
Risk of bias in studies	19	Present evaluation of risk of bias and concerns regarding applicability for each study.	4
Results of individual studies	20	For each analysis in each study (e.g., unique combination of index test, reference standard, and positivity threshold) report 2x2 data (TP, FP, FN, TN) with estimates of diagnostic accuracy and confidence intervals, ideally with a forest or receiver operator characteristic (ROC) plot.	4-6
Synthesis of results	22	Describe test accuracy, including variability; if meta-analysis was done, include results and confidence intervals.	4-6
Additional analysis	23	Give results of additional analyses, if done (e.g., sensitivity or subgroup analyses, meta-regression; analysis of index test: failure rates, proportion of inconclusive results, adverse events).	4-6
**DISCUSSION**			
Summary of evidence	24	Summarize the main findings including the strength of evidence.	6-7
Limitations	25	Discuss limitations from included studies (e.g., risk of bias and concerns regarding applicability) and from the review process (e.g., incomplete retrieval of identified research).	10-11
Conclusions	26	Provide a general interpretation of the results in the context of other evidence. Discuss implications for future research and clinical practice (e.g., the intended use and clinical role of the index test).	11
**FUNDING**			
Funding	26	For the systematic review, describe the sources of funding and other support and the role of the funders.	14

Salameh, Jean-Paul et al. "Preferred reporting items for systematic review and meta-analysis of diagnostic test accuracy studies (PRISMA-DTA): explanation, elaboration, and checklist." *BMJ (Clinical research ed.)* vol. 370 m2632. 14 Aug. 2020, doi:10.1136/bmj.m2632

Supplementary Table 1 Search strategy in PubMed, Embase and Web of Science.

Database	Search strategy
PubMed	("Artificial Intelligence"[Mesh] OR "Machine Learning"[Mesh] OR "Deep Learning"[Mesh] OR "artificial intelligence"[Title/Abstract] OR "machine learning"[Title/Abstract] OR "deep learning"[Title/Abstract] OR "AI"[Title/Abstract] OR "ML"[Title/Abstract] OR "DL"[Title/Abstract]) AND ("Colorectal Neoplasms"[Mesh] OR "Rectal Neoplasms"[Mesh] OR "Colonic Neoplasms"[Mesh] OR "colorectal cancer*"[Title/Abstract] OR "colorectal tumor*"[Title/Abstract] OR "colorectal tumour*"[Title/Abstract] OR "colorectal neoplasm*"[Title/Abstract] OR "colorectal carcinoma*"[Title/Abstract] OR "CRC"[Title/Abstract] OR "colon cancer*"[Title/Abstract] OR "rectal cancer*"[Title/Abstract] OR "bowel cancer*"[Title/Abstract]) AND ("Microsatellite Instability"[Mesh] OR "microsatellite instability"[Title/Abstract] OR "MSI"[Title/Abstract] OR "microsatellite unstable"[Title/Abstract] OR "deficient mismatch repair"[Title/Abstract] OR "dMMR"[Title/Abstract] OR "MMR deficient"[Title/Abstract])
Embase	('artificial intelligence'/exp OR 'machine learning'/exp OR 'deep learning'/exp OR 'artificial intelligence':ti,ab OR 'machine learning':ti,ab OR 'deep learning':ti,ab OR 'AI':ti,ab OR 'ML':ti,ab OR 'DL':ti,ab) AND ('colorectal cancer'/exp OR 'colorectal tumor':ti,ab OR 'colorectal tumour':ti,ab OR 'colorectal neoplasm':ti,ab OR 'colorectal carcinoma':ti,ab OR 'CRC':ti,ab OR 'colon cancer':ti,ab OR 'rectal cancer':ti,ab OR 'bowel cancer':ti,ab) AND ('microsatellite instability'/exp OR 'microsatellite instability':ti,ab OR 'MSI':ti,ab OR 'microsatellite unstable':ti,ab OR 'deficient mismatch repair':ti,ab OR 'dMMR':ti,ab OR 'MMR deficient':ti,ab)
Web of Science	TS=("Artificial Intelligence" OR "Machine Learning" OR "Deep Learning" OR "artificial intelligence" OR "machine learning" OR "deep learning" OR "AI" OR "ML" OR "DL") AND TS=("Colorectal Neoplasms" OR "Rectal Neoplasms" OR "Colonic Neoplasms" OR "colorectal cancer" OR "colorectal tumor" OR "colorectal tumour" OR "colorectal neoplasm" OR "colorectal carcinoma" OR "CRC" OR "colon cancer" OR "rectal cancer" OR "bowel cancer") AND TS=("Microsatellite Instability" OR "microsatellite instability" OR "MSI" OR "microsatellite unstable" OR "deficient mismatch repair" OR "dMMR" OR "MMR deficient")

Supplementary Table 2 Revised QUADAS-2 tool for the included studies.

Author	Risk of bias				Applicability concerns		
	Patient selection	Index test	Reference standard	Analysis	Patient selection	Index test	Reference standard
Hezi et al.2024	U	L	L	U	L	L	L
Gustav et al.2024	L	L	L	L	L	L	L
Tong et al.2023	U	L	L	U	L	L	L
Saillard et al.2023	L	L	L	U	L	L	L
Niehues et al.2023	U	L	L	U	L	L	L
Guo et al.2023	U	L	L	U	L	L	L
Gerwert et al.2023	U	L	L	U	L	L	L
Chang et al.2023	U	L	L	U	L	L	L
Qiu et al.2022	U	L	L	U	L	L	L
Wu et al.2022	U	L	L	L	L	L	L
Guo et al.2022	U	L	L	U	L	L	L
Fujii et al.2022	U	L	L	U	L	L	L
Echle et al.2022	U	L	L	U	L	L	L
Kather et al.2019	U	L	L	U	L	L	L
Echle et al.2020	U	L	L	U	L	L	L
Cao et al.2020	U	L	L	U	L	L	L
Yamashita et al.2021	U	L	L	U	L	L	L
Krause et al.2021	U	L	L	U	L	L	L
Lee et al.2021	U	L	L	U	L	L	L

L low; H high; U unclear.

a. **Patient selection**

- Low risk: No inappropriate exclusions.
- High risk: Inappropriate exclusions (e.g., excluding patients under 18, restricting to specific treatments/subtypes/timeframes).
- Unclear: Insufficient information to assess exclusions.

b. **Index test**

- Low risk: Detailed model training/validation processes provided or cited from a prior publication with full modification details.
- High risk: Only model name reported without key training details (e.g., algorithm unspecified).
- Unclear: Model name given but training process indeterminable.

c. **Reference standard**

- Low risk: Final diagnosis made blinded to AI results.
- High risk: AI results used to inform final diagnosis.
- Unclear: Blinding status unreported.

d. **Analysis**

- Low risk: All enrolled participants included in meta-analysis.
- High risk: Selective exclusion of participants/subgroups.
- Unclear: Inclusion criteria inadequately described.

e. **Patient selection**

- Low risk: Study population aligns with meta-analysis inclusion criteria.
- High risk: Study includes ineligible patients per meta-analysis criteria.
- Unclear: Population eligibility unclear.

f. **Index test**

- Low risk: AI definition matches meta-analysis criteria.
- High risk: AI definition partially deviates from criteria.
- Unclear: AI definition adequacy unverifiable.

g. **Reference standard**

- Low risk: Reference standard aligns with meta-analysis criteria.
- High risk: Reference standard inconsistently applied.
- Unclear: Reference standard details missing.

Supplementary Table 3 Leave-one-out sensitivity analysis for sensitivity and specificity of internal validation sets based on patient-based analysis and image-based analysis.

Omitting study	Sensitivity(95%CI)	I^2 for sensitivity	Specificity	I^2 for specificity
**Patient-based analysis**				
Omitting Hezi et al.	0.89 (0.82 - 0.93)	90.14%	0.86 (0.76 - 0.93)	99.35%
Omitting Gustav et al.	0.90 (0.85 - 094)	85.33%	0.86 (0.75 - 0.92)	99.29%
Omitting Tong et al.	0.87(0.80 - 0.92)	88.09%	0.84 (0.75 - 0.90)	99.18%
Omitting Niehues et al.	0.86 (0.80 - 0.96)	79.25%	0.89 (0.83 - 0.93)	96.26%
Omitting Gerwert et al.	0.89 (0.82 - 0.93)	90.36%	0.87 (0.77 - 0.93)	99.36%
Omitting Wu et al.	0.88(0.81 - 0.93)	88.17%	0.84(0.75 - 0.91)	99.17%
Omitting Echle et al.	0.89(0.82 - 0.93)	91.05%	0.87(0.76 - 0.93)	99.32%
Omitting Krause et al.	0.89(0.83 - 0.93)	91.14%	0.87(0.78 - 0.93)	99.43%
**Image-based analysis**				
Omitting Chang et al.	0.80 (0.71 - 0.87)	17.16%	0.75(0.72 - 0.78)	0%
Omitting Qiu et al.	0.84 (0.78 - 0.89)	90.42%	0.84(0.73 - 0.92)	96.74%
Omitting Guo et al.	0.83(0.78 - 0.87)	90.12%	0.82(0.71 - 0.90)	97.17%
Omitting Fujii et al. (second stage)	0.81(0.76 - 0.85)	90.83%	0.82(0.7 - 0.90)	97.17%
Omitting Fujii et al. (2.5^{th} stage)	0.81(0.76 - 0.85)	88.87%	0.83(0.72 - 0.91)	96.96%
Omitting Cao et al.	0.78(0.71 - 0.84)	92.31%	0.84(0.72 - 0.91)	96.60%

Supplementary Table 4 Leave-one-out sensitivity analysis for sensitivity and specificity of external validation sets based on patient-based analysis and image-based analysis.

Omitting study	Sensitivity	I^2 for sensitivity	Specificity	I^2 for specificity
**Patient-based analysis**				
Omitting Gustav et al.	0.93 (0.88 - 0.96)	96.62%	0.72 (0.57 - 0.83)	99.62%
Omitting Tong et al.	0.93 (0.89 - 0.96)	96.32%	0.68 (0.54 - 0.80)	99.60%
Omitting Niehues et al.	0.93 (0.88 - 0.96)	96.71%	0.70 (0.55 - 0.82)	99.59%
Omitting Guo et al.	0.93 (0.88 - 0.96)	96.48%	0.70 (0.55 - 0.82)	99.60%
Omitting Wu et al.(surgical)	0.93 (0.88 - 0.96)	96.25	0.68 (0.54 - 0.80)	99.57%
Omitting Wu et al.(biopsy)	0.93 (0.88 - 0.96)	96.04%	0.68 (0.54 - 0.80)	99.56%
Omitting Echle et al. (DACHS)	0.92 (0.87 - 0.95)	96.02%	0.72 (0.58 - 0.83)	99.56%
Omitting Echle et al. (DUESSEL)	0.93 (0.88 - 0.96)	96.55%	0.71 (0.56 - 0.82)	99.62%
Omitting Echle et al. (MECC)	0.93 (0.88 - 0.96)	96.59%	0.72 (0.59 - 0.83)	99.62%
Omitting Echle et al. (MUNICH)	0.93 (0.88 - 0.96)	96.54%	0.71 (0.56 - 0.82)	99.62%
Omitting Echle et al. (NLCS)	0.92 (0.87 - 0.95)	96.42%	0.71 (0.57 - 0.83)	99.62%
Omitting Echle et al. (QUASAR)	0.92 (0.87 - 0.95)	95.93%	0.72 (0.58 - 0.82)	99.60%
Omitting Echle et al. (TCGA)	0.93 (0.88 - 0.96)	96.53%	0.71 (0.56 - 0.82)	99.61%
Omitting Echle et al. (UMM)	0.93 (0.88 - 0.96)	96.36%	0.70 (0.55 - 0.82)	99.60%
Omitting Echle et al. (YORKSHIRE)	0.92 (0.87 - 0.95)	96.10%	0.73 (0.60 - 0.83)	99.60%
Omitting Echle et al.(biopsy)	0.91 (0.87 - 0.94)	94.39%	0.74 (0.61 - 0.83)	99.47%
Omitting Kather et al.	0.93 (0.88 - 0.96)	96.28%	0.70 (0.55 - 0.81)	99.59%
Omitting Echle et al.(surgical)	0.93 (0.88 - 0.96)	96.37%	0.69 (0.54 - 0.81)	99.56%
Omitting Echle et al.(biopsy)	0.93 (0.89 - 0.96)	95.75%	0.71 (0.56 - 0.82)	99.61%
Omitting Yamashita et al.	0.93 (0.89 - 0.96)	96.44%	0.71 (0.56 - 0.82)	99.61%
Omitting Lee et al.	0.92 (0.88 - 0.96)	96.45%	0.69 (0.54 - 0.80)	99.59%
**Image-based analysis**				

Omitting Saillard et al. (MAPTH-DP200)	0.84 (0.65 - 0.93)	85.66%	0.57 (0.40 - 0.73)	96.86%
Omitting Saillard et al. (MAPTH-UFS)	0.70 (0.63 - 0.76)	31.30%	0.57 (0.40 - 0.73)	96.92%
Omitting Chang et al.	0.82 (0.60 - 0.93)	91.64%	0.57 (0.40 - 0.73)	97.62
Omitting Cao et al.	0.82 (0.58 - 0.93)	88.57%	0.46 (0.43 - 0.49)	100.0%

Supplementary Figure 1 Forest plot of deep learning algorithms for identifying microsatellite instability-high in colorectal cancer using whole slide images in the external validation set of patient-based analysis. Squares represent the sensitivity and specificity of each study, while horizontal bars indicate the 95% confidence intervals. This figure was generated using Stata 15.1 software.

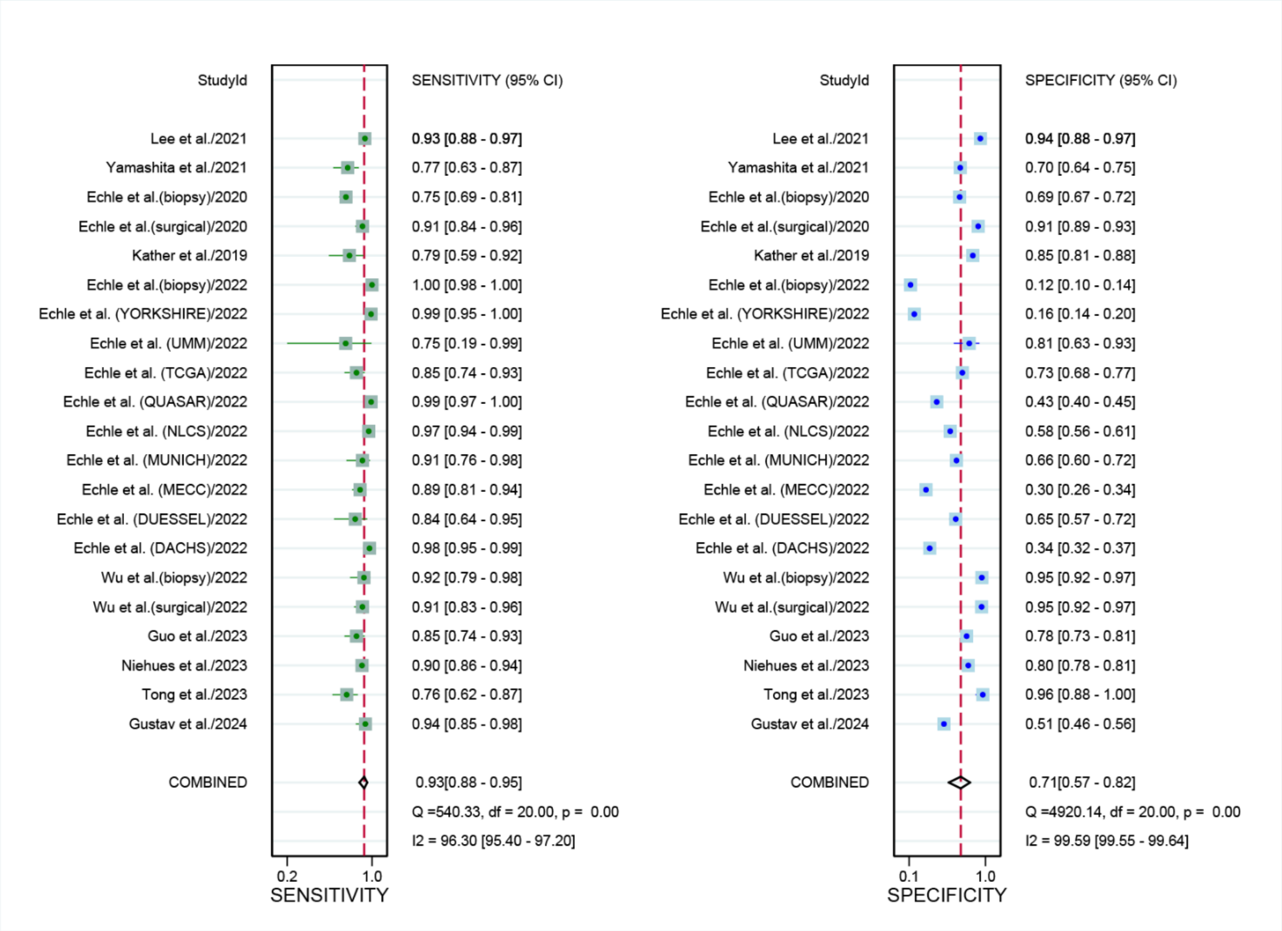

Supplementary Figure 2 Summary receiver operating characteristic (SROC) curves of deep learning algorithms for identifying microsatellite instability-high in colorectal cancer using whole slide images in the external validation set of patient-based (a) and image-based (b). This figure was generated using Stata 15.1 software.

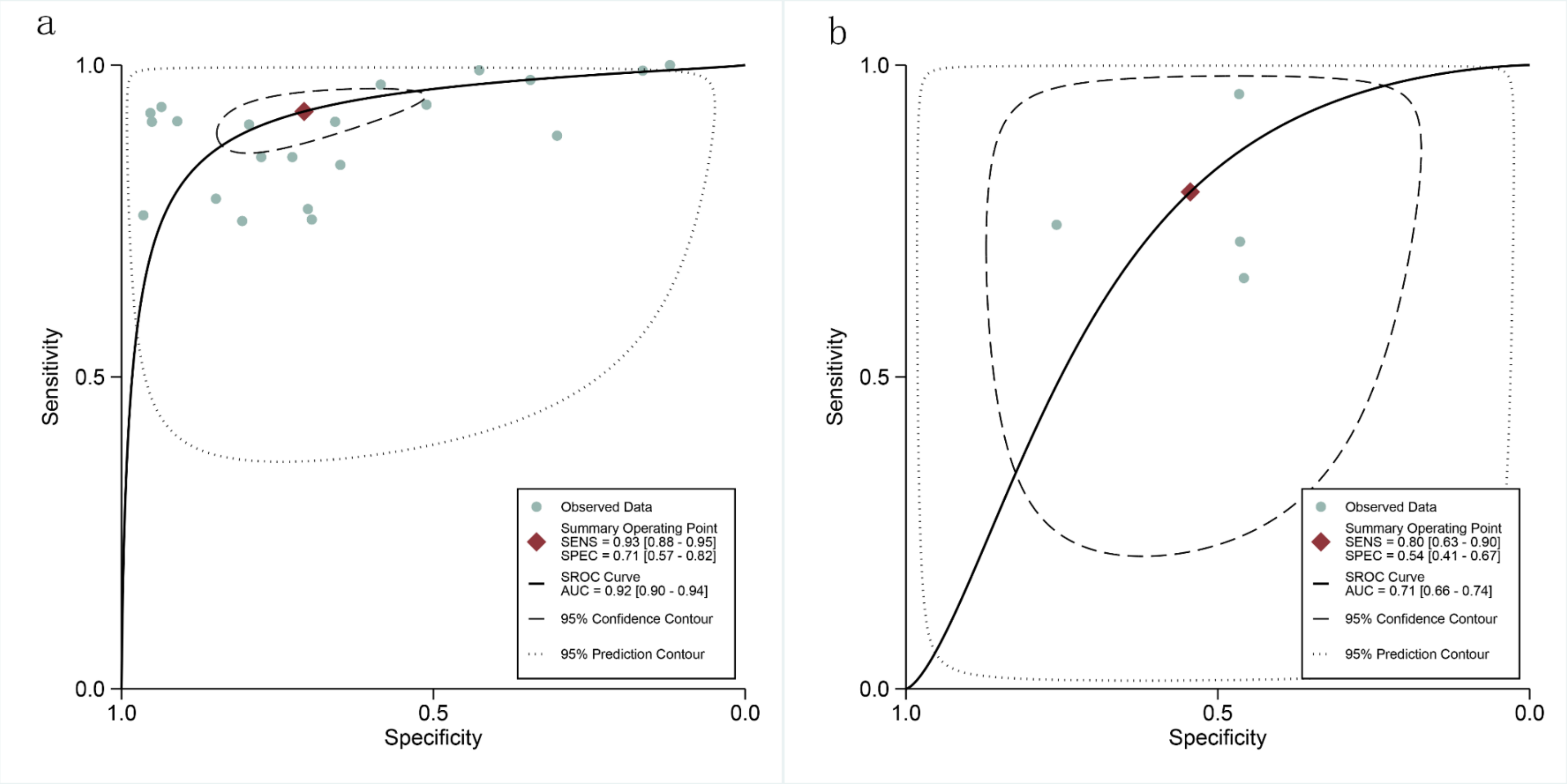

Supplementary Figure 3 Fagan's nomogram for deep learning algorithms in identifying microsatellite instability-high in colorectal cancer using whole slide images in the external validation set of patient-based (a) and image-based (b). This figure was generated using Stata 15.1 software.

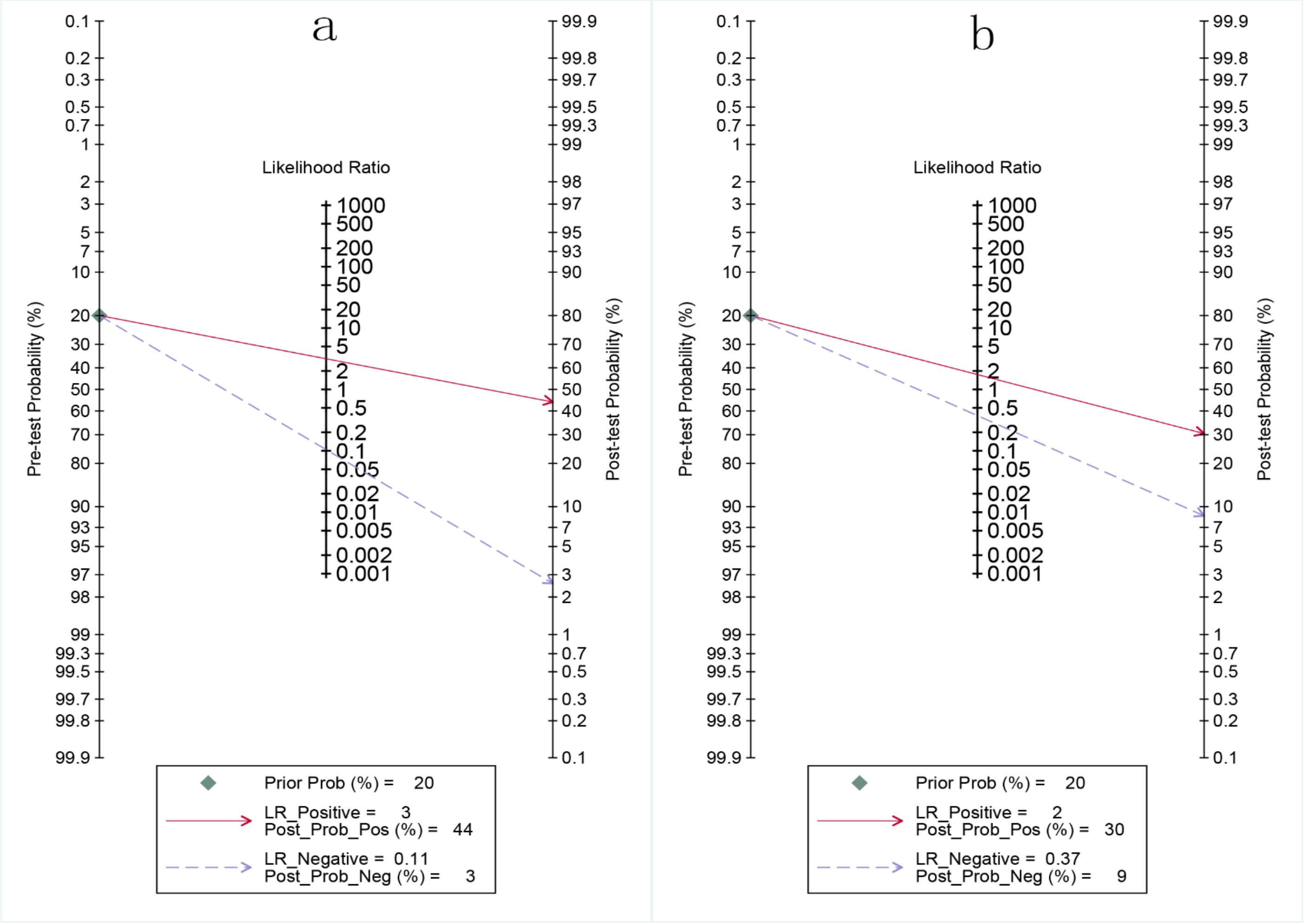

Supplementary Figure 4 Forest plot of deep learning algorithms for identifying microsatellite instability-high in colorectal cancer using whole slide images in the external validation set of image-based analysis. Squares represent the sensitivity and specificity of each study, while horizontal bars indicate the 95% confidence intervals. This figure was generated using Stata 15.1 software.

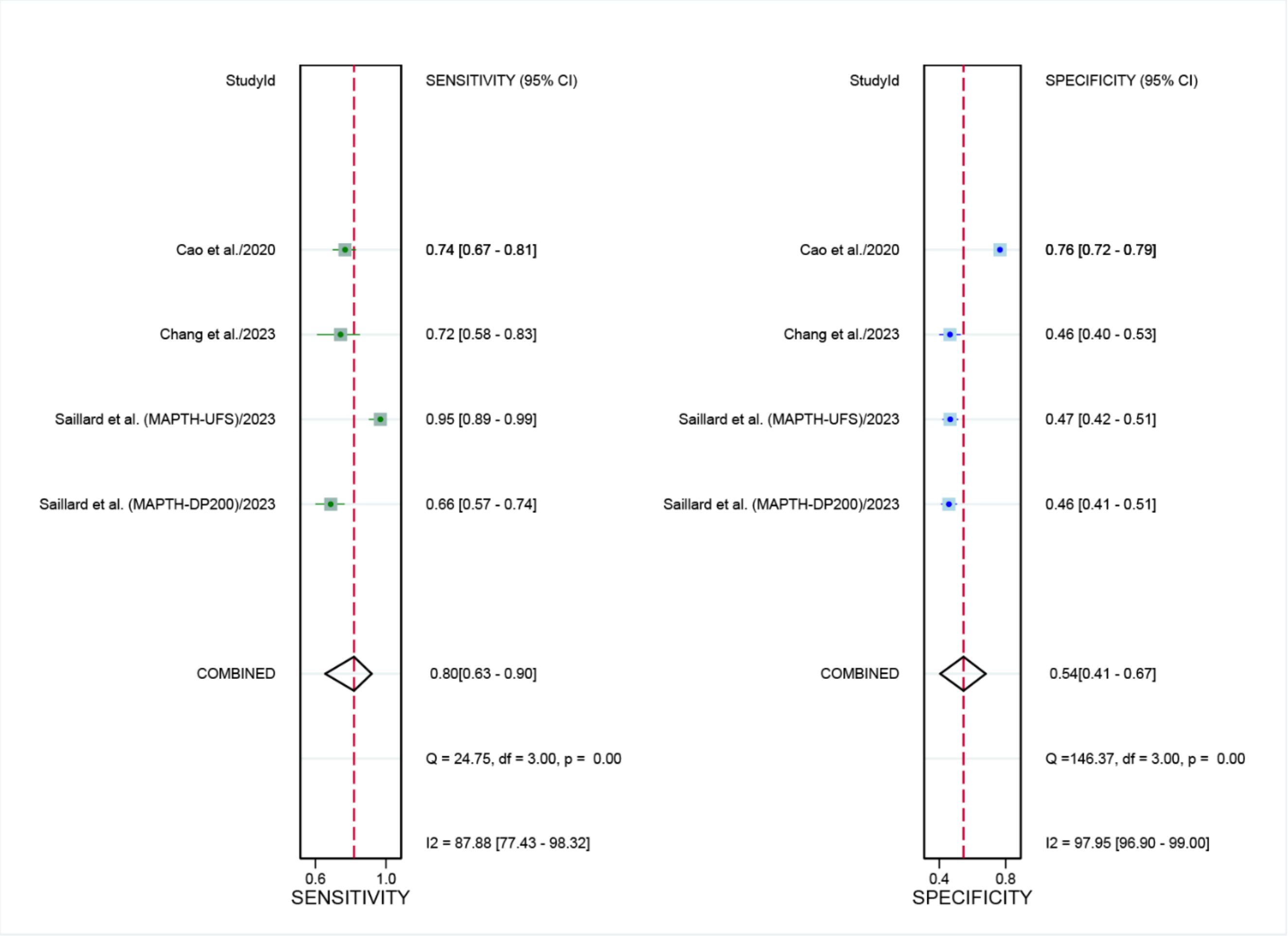

Supplementary Figure 5 Deek's funnel plot of diagnostic performance of deep learning algorithms for identifying microsatellite instability-high in colorectal cancer using whole slide images in the external validation set of patient-based (a) and image-based (b). P < 0.05 was considered significant. This figure was generated using Stata 15.1 software.

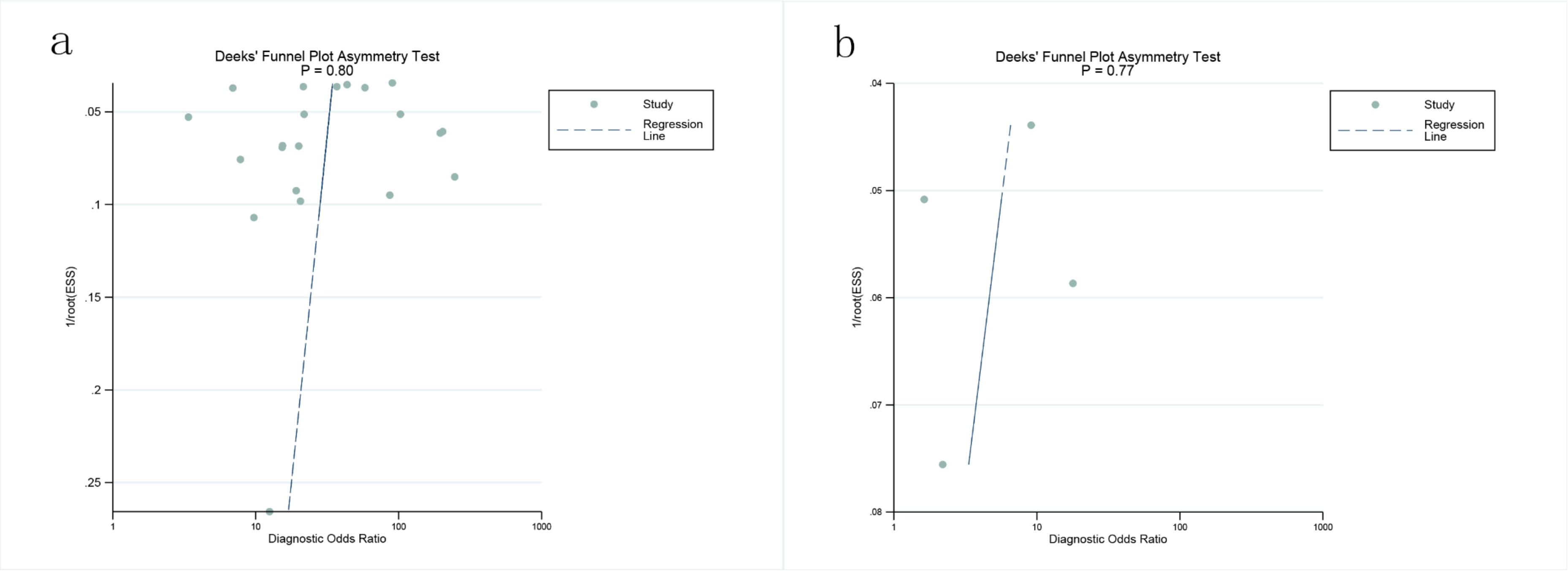

Supplement: Supplementary file 1 — Supplementary materials [file 41746_2025_1848_MOESM1_ESM.pdf]
